# Supplementary material for: Multi‐dataset identification of innovative feature genes and molecular mechanisms in keratoconus
Source: J Cell Mol Med. 2024 Sep 19;28(18):e70079. doi: 10.1111/jcmm.70079 (PMC11412914; doi:10.1111/jcmm.70079)
Supplement: Supplementary file 2 — Material S2. [file JCMM-28-e70079-s002.zip › 7.GSEA analysis/1.ARL11/KEGG_GLYCOSAMINOGLYCAN_BIOSYNTHESIS_HEPARAN_SULFATE.html]

Details for gene set KEGG\_GLYCOSAMINOGLYCAN\_BIOSYNTHESIS\_HEPARAN\_SULFATE[GSEA]

|  || Dataset | ARL11.group.cls#h\_versus\_l |
| Phenotype | group.cls#h\_versus\_l |
| Upregulated in class | h |
| GeneSet | KEGG\_GLYCOSAMINOGLYCAN\_BIOSYNTHESIS\_HEPARAN\_SULFATE |
| Enrichment Score (ES) | 0.27993718 |
| Normalized Enrichment Score (NES) | 0.9143103 |
| Nominal p-value | 0.5838384 |
| FDR q-value | 1.0 |
| FWER p-Value | 1.0 |
Table: GSEA Results Summary

  

Fig 1: Enrichment plot: KEGG\_GLYCOSAMINOGLYCAN\_BIOSYNTHESIS\_HEPARAN\_SULFATE      
 Profile of the Running ES Score & Positions of GeneSet Members on the Rank Ordered List

  

| SYMBOL | TITLE | RANK IN GENE LIST | RANK METRIC SCORE | RUNNING ES | CORE ENRICHMENT || 1 | HS3ST3A1 | na | 211 | 0.572 | 0.2122 | Yes |
| 2 | NDST4 | na | 2210 | 0.281 | 0.2620 | Yes |
| 3 | EXTL1 | na | 4931 | 0.168 | 0.2478 | Yes |
| 4 | HS3ST2 | na | 7338 | 0.105 | 0.2184 | Yes |
| 5 | NDST1 | na | 7472 | 0.102 | 0.2536 | Yes |
| 6 | HS3ST5 | na | 7829 | 0.096 | 0.2799 | Yes |
| 7 | HS6ST3 | na | 8883 | 0.077 | 0.2789 | No |
| 8 | HS6ST1 | na | 10430 | 0.055 | 0.2551 | No |
| 9 | EXTL3 | na | 10656 | 0.052 | 0.2684 | No |
| 10 | B3GALT6 | na | 11318 | 0.044 | 0.2660 | No |
| 11 | XYLT2 | na | 12163 | 0.035 | 0.2549 | No |
| 12 | B3GAT3 | na | 12696 | 0.030 | 0.2509 | No |
| 13 | NDST2 | na | 18521 | -0.014 | 0.0883 | No |
| 14 | B4GALT7 | na | 19329 | -0.019 | 0.0724 | No |
| 15 | EXTL2 | na | 20686 | -0.029 | 0.0444 | No |
| 16 | B3GAT2 | na | 21892 | -0.039 | 0.0243 | No |
| 17 | EXT2 | na | 22470 | -0.045 | 0.0248 | No |
| 18 | EXT1 | na | 23347 | -0.054 | 0.0201 | No |
| 19 | HS3ST1 | na | 23488 | -0.056 | 0.0373 | No |
| 20 | GLCE | na | 24593 | -0.071 | 0.0325 | No |
| 21 | XYLT1 | na | 24720 | -0.072 | 0.0564 | No |
| 22 | HS2ST1 | na | 25536 | -0.083 | 0.0648 | No |
| 23 | B3GAT1 | na | 25591 | -0.084 | 0.0953 | No |
| 24 | NDST3 | na | 26185 | -0.093 | 0.1138 | No |
| 25 | HS6ST2 | na | 26636 | -0.101 | 0.1394 | No |
| 26 | HS3ST3B1 | na | 31654 | -0.246 | 0.0886 | No |
Table: GSEA details [plain text format]

  

Fig 2: KEGG\_GLYCOSAMINOGLYCAN\_BIOSYNTHESIS\_HEPARAN\_SULFATE      
 Blue-Pink O' Gram in the Space of the Analyzed GeneSet

  

Fig 3: KEGG\_GLYCOSAMINOGLYCAN\_BIOSYNTHESIS\_HEPARAN\_SULFATE: Random ES distribution      
 Gene set null distribution of ES for **KEGG\_GLYCOSAMINOGLYCAN\_BIOSYNTHESIS\_HEPARAN\_SULFATE**
